# Cancers in the first-degree relatives of children with brain tumours

**DOI:** 10.1054/bjoc.2000.1252

**Published:** 2000-07-03

**Authors:** K Hemminki, X Li, P Vaittinen, C Dong

**Affiliations:** Department of Biosciences at Novum, Karolinska Institute, Huddinge, 141 57, Sweden

**Keywords:** astrocytoma, medulloblastoma, ependymoma, meningioma, second cancer

## Abstract

We used the nationwide Swedish Family-Cancer Database with 2060 childhood brain tumours diagnosed in the period 1958–1996 to analyse the risk of this tumour by parental cancers and in siblings of childhood brain tumour probands. Groups of patients were compared by calculating standardized incidence ratios (SIRs) for brain tumours in offspring. 1.3% of brain tumour patients had a parent with nervous system cancer; SIRs were 2.4 and 1.88 for diagnostic ages < 5 and < 15 years, respectively. The data showed distinct patterns of familial risks for childhood brain tumours, the SIR was 10.26 for brain astrocytoma given a parent with meningioma. Parental colon cancer was associated with offspring ependymoma (SIR 3.70), and parental salivary gland cancers with offspring medulloblastoma (SIR 13.33, but two cases only). SIR for sibling nervous system cancer from childhood brain tumour probands was 3.55 up to age 61. © 2000 Cancer Research Campaign


*British Journal of Cancer* (2000) **83**(3), 407–411
© 2000 Cancer Research Campaign
doi: 10.1054/ bjoc.2000.1252, available online at http://www.idealibrary.com on IDEAL®

### Cancers in the first-degree relatives of children with brain tumours

**K Hemminki, X Li, P Vaittinen and C Dong**

Department of Biosciences at Novum, Karolinska Institute, 141 57 Huddinge, Sweden

**Summary** We used the nationwide Swedish Family-Cancer Database with 2060 childhood brain tumours diagnosed in the period 1958–1996 to analyse the risk of this tumour by parental cancers and in siblings of childhood brain tumour probands. Groups of patients were compared by calculating standardized incidence ratios (SIRs) for brain tumours in offspring. 1.3% of brain tumour patients had a parent with nervous system cancer; SIRs were 2.4 and 1.88 for diagnostic ages < 5 and < 15 years, respectively. The data showed distinct patterns of familial risks for childhood brain tumours, the SIR was 10.26 for brain astrocytoma given a parent with meningioma. Parental colon cancer was associated with offspring ependymoma (SIR 3.70), and parental salivary gland cancers with offspring medulloblastoma (SIR 13.33, but two cases only). SIR for sibling nervous system cancer from childhood brain tumour probands was 3.55 up to age 61. © 2000 Cancer Research Campaign

**Keywords**: astrocytoma; medulloblastoma; ependymoma; meningioma; second cancer

Nervous system cancers and acute leukaemias are the most common malignancies in children (0–14) (Draper et al, 1994; IARC, 1998; Linet et al, 1999). About 80% of nervous system cancers are brain tumours, the incidence of which is highest in the Nordic countries being about 5 per 100 000 per year in Sweden (Hemminki et al, 1999; Hjalmars et al, 1999). In spite of extensive studies, the causes of childhood brain cancer remain elusive, ionizing radiation and genetic predisposition being the only established risk factors (Bondy et al, 1991; Draper et al, 1994; Zahm and Devesa, 1995; Little et al, 1998; Little, 1999; Salminen et al, 1999; Kleihues and Cavenee, 2000). The incidence of brain tumour, particularly in young children (< 5 years) has increased moderately in most industrialized countries from the 1960s to the 1990s (Draper et al, 1994; IARC, 1998; Hemminki et al, 1999; Linet et al, 1999). Brain tumour is a component of certain rare cancer syndromes such as Li-Fraumeni, neurofibromatosis 1 and 2, von Hippel-Lindau and Gorlin (Li, 1995; Fearon, 1997; Huson, 1998). However, these conditions are likely to explain only a small proportion of childhood brain tumours; only 2% based on a review of records (Narod et al, 1991). No increased risk of brain tumour was observed in parents of childhood brain tumour patients (Olsen et al, 1995), though among offspring of survivors of childhood brain tumours the relative risk was 2.0 and almost significant (Sankila et al, 1998).

Here we analyse the risk of childhood brain tumours of defined histological types by parental cancer using the nationwide Swedish Family-Cancer Database (Hemminki et al., 1998; Hemminki and Vaittinen, 1999; Hemminki and Dong, 2000). Risks to siblings are also determined. The Database offers unique possibilities for reliable estimation of familial risks, because the data on family relationships and cancers were obtained from registered sources of practically complete coverage (Hemminki and Vaittinen, 1998a; Hemminki et al, 1998). Here we use the 1999 update of the nationwide Swedish Family-Cancer Database, now covering 9.6 million individuals in a country of 8.8 million population.

*Received 18 February 2000*
*Revised 15 March 2000*
*Accepted 21 March 2000*

*Correspondence to:* K Hemminki

### SUBJECTS AND METHODS

The Swedish Family-Cancer Database includes persons, (‘offspring’) born in Sweden after 1934 and their biological parents (Hemminki & Vaittinen, 1998b; Hemminki et al, 1998). Cancers were retrieved from the nationwide Swedish Cancer Registry from years 1958 to 1996. A 4-digit diagnostic code according to the 7th revision of the International Classification of Diseases (ICD-7) was used. ICD code 1930 was used for brain tumours. The histological classification of brain tumour was used to define astrocytoma, pathological anatomic diagnosis (PAD) codes 471–476, medulloblastoma (436), ependymoma (481–486) meningioma (461–466) and other subtypes.

There were 3 families with two siblings presenting with childhood brain tumours and no families with three or more affected siblings. In the analysis of parent–offspring risks, the siblings were treated independently, i.e., a sibship of two affected individuals contributed two affected parent–offspring pairs. In the analysis of sibling risks, person-years were calculated for families of one childhood brain tumour proband. Standardized incidence ratios (SIRs) were calculated as the ratio of observed (O) to expected (E) number of cases. The expected numbers were calculated from 5-year-age- and tumour type-standardized rates (Esteve et al, 1994). Confidence intervals (95% CI) were calculated assuming a Poisson distribution (Esteve et al, 1994).

### RESULTS

The Family-Cancer Database included 752 and 2060 cases of childhood brain cancer, diagnosed before age 5 and before age 15,

**Table 1** Histopathological distribution of childhood brain tumour in the Family-Cancer Database

| Histopathological type | Age at diagnosis < 5 Cases | < 5 % | < 15 Cases | < 15 % |
|---|---|---|---|---|
| Astrocytoma | 333 | 44.3 | 1001 | 48.6 |
| Medulloblastoma | 129 | 17.1 | 316 | 15.3 |
| Ependymoma | 114 | 15.1 | 210 | 10.2 |
| Craniopharyngioma | 20 | 2.7 | 77 | 3.7 |
| Pineal tumours | 5 | 0.7 | 27 | 1.3 |
| Meningioma | 9 | 1.2 | 22 | 1.1 |
| Others | 142 | 18.9 | 407 | 19.8 |
| All | 752 | 100 | 2060 | 100 |

**Table 2** SIR for childhood brain tumour by parental cancer

| Parental cancer | Age at diagnosis < 5 O | E | SIR | 95% CI | | < 15 O | E | SIR | 95% CI | |
|---|---|---|---|---|---|---|---|---|---|---|
| No cancer | 684 | | | | | 1818 | | | | |
| Oral | 3 | 1.74 | 1.72 | 0.33 | 4.23 | 9 | 7.3 | 1.23 | 0.56 | 2.17 |
| Salivary glands | | | | | | 2 | 1.00 | 2.00 | 0.19 | 5.73 |
| Stomach | 1 | 2.09 | 0.48 | 0.00 | 1.88 | 8 | 10.3 | 0.78 | 0.33 | 1.41 |
| Colon | 4 | 4.08 | 0.98 | 0.26 | 2.18 | 20 | 18.43 | 1.09 | 0.66 | 1.61 |
| Rectum | 2 | 2.48 | 0.81 | 0.08 | 2.31 | 10 | 11.18 | 0.89 | 0.43 | 1.53 |
| Liver | 2 | 1.46 | 1.37 | 0.13 | 3.93 | 4 | 7.14 | 0.56 | 0.15 | 1.24 |
| Lung | 3 | 4.98 | 0.60 | 0.11 | 1.48 | 11 | 21.83 | 0.50 | 0.25 | 0.85 |
| Breast | 7 | 12.7 | 0.55 | 0.22 | 1.04 | 31 | 47.35 | 0.65 | 0.44 | 0.91 |
| Cervix | 3 | 2.79 | 1.08 | 0.20 | 2.64 | 9 | 10.52 | 0.86 | 0.39 | 1.51 |
| Endometrium | 3 | 1.98 | 1.52 | 0.29 | 3.71 | 10 | 9.02 | 1.11 | 0.53 | 1.90 |
| Ovary | 4 | 2.25 | 1.78 | 0.46 | 3.95 | 7 | 9.07 | 0.77 | 0.31 | 1.45 |
| Prostate | 4 | 5.46 | 0.73 | 0.19 | 1.63 | 21 | 28.67 | 0.73 | 0.45 | 1.08 |
| Kidney | | | | | | 9 | 10.07 | 0.89 | 0.41 | 1.57 |
| Bladder | 3 | 2.97 | 1.01 | 0.19 | 2.48 | 7 | 13.1 | 0.53 | 0.21 | 1.00 |
| Melanoma | 5 | 4.49 | 1.11 | 0.35 | 2.30 | 17 | 14.59 | 1.17 | 0.68 | 1.78 |
| Skin | 2 | 1.42 | 1.41 | 0.13 | 4.04 | 8 | 6.68 | 1.20 | 0.51 | 2.17 |
| Nervous system | 10 | 3.94 | **2.54** | **1.21** | **4.35** | 26 | 13.85 | **1.88** | **1.23** | **2.67** |
| Thyroid gland | 2 | 1.32 | 1.52 | 0.14 | 4.34 | 5 | 4.34 | 1.15 | 0.36 | 2.38 |
| Connective tissue | 2 | 0.71 | 2.82 | 0.27 | 8.07 | 5 | 2.57 | 1.95 | 0.61 | 4.02 |
| Lymphoma | 1 | 3.37 | 0.30 | 0.00 | 1.16 | 8 | 12.41 | 0.64 | 0.28 | 1.17 |
| Leukaemia | 2 | 2.02 | 0.99 | 0.09 | 2.84 | 6 | 8.20 | 0.73 | 0.26 | 1.43 |
| Other sites | 7 | 5.61 | 1.25 | 0.49 | 2.34 | 23 | 28.26 | 0.81 | 0.52 | 1.18 |

Bold type: 95% CI does not include 1.00

respectively (Table 1). The most common histological types were astrocytoma, medulloblastoma and ependymoma; less common types were craniopharyngiomas, pineal tumours and meningiomas. Together these accounted for 82% of brain tumours till age 5 and 80% till age 15.

The SIR for childhood brain tumour was analysed by parental cancer in Table 2. Paternal and maternal cancer sites were also analysed separately but because there were no large differences in SIRs for the common sites, the data in Table 2 were combined for both parents. SIR by parental nervous system cancer was 2.54 for offspring diagnosed before age 5 and 1.88 for offspring diagnosed before age 15. There were no other parental sites that associated with the risk in offspring. Comparing the numbers of cases from Tables 1 and 2, we note that 1.3% (10/752, diagnosis < 5 years and 26/2060, diagnosis < 15 years) of childhood brain tumour cases had a parent with nervous system cancer.

Analysis was carried out by specific subtype of brain tumour in offspring (Table 3). The most common type, astrocytoma, accounted for most of the brain tumours where parents were affected by nervous system cancer. SIR for astrocytoma was 2.67 when diagnosed < 15 years and 5.14 when diagnosed < 5 years. Parental salivary gland cancers increased the SIR of medulloblastoma to 13.33 but there were only two cases (95% CI, 1.26–38.22). Ependymoma showed a SIR of 3.70 when parents had colon cancer. Two ependymoma patients had a mother with endometrial cancer (SIR 2.17, 95% CI, 0.20–6.23).

We analysed offspring astrocytomas by subtypes of parental nervous system tumours. SIR for astrocytoma by parental brain

 *© 2000 Cancer Research Campaign*

**Table 3** SIR for childhood brain tumour subtypes (diagnosis < 15 years) by parental cancer

| Parental cancer | Astrocytoma | | | | | Medulloblastoma | | | | | Ependymoma | | | | |
|---|---|---|---|---|---|---|---|---|---|---|---|---|---|---|---|
| | O | E | SIR | 95% CI | | O | E | SIR | 95% CI | | O | E | SIR | 95% CI | |
| No cancer | 891 | | | | | 284 | | | | | 177 | | | | |
| Oral | 7 | 3.55 | 1.97 | 0.78 | 3.70 | | | | | | | | | | |
| Salivary glands | | | | | | 2 | 0.15 | **13.33** | **1.26** | **38.22** | | | | | |
| Stomach | 4 | 5.01 | 0.80 | 0.21 | 1.77 | | | | | | | | | | |
| Colon | 3 | 8.96 | 0.33 | 0.06 | 0.82 | 4 | 2.83 | 1.41 | 0.37 | 3.14 | **7** | 1.90 | **3.70** | **1.47** | **6.96** |
| Rectum | 5 | 5.44 | 0.92 | 0.29 | 1.90 | | | | | | | | | | |
| Liver | 3 | 3.47 | 0.86 | 0.16 | 2.12 | | | | | | | | | | |
| Lung | 7 | 10.62 | 0.66 | 0.26 | 1.24 | 2 | 3.35 | 0.6 | 0.06 | 1.71 | | | | | |
| Breast | 16 | 23.03 | 0.69 | 0.40 | 1.08 | 5 | 7.26 | 0.69 | 0.22 | 1.42 | 2 | 4.85 | 0.41 | 0.04 | 1.18 |
| Cervix | 3 | 5.12 | 0.59 | 0.11 | 1.44 | | | | | | | | | | |
| Endometrium | 4 | 4.39 | 0.91 | 0.24 | 2.02 | | | | | | 2 | 0.92 | 2.17 | 0.20 | 6.23 |
| Ovary | 4 | 4.41 | 0.91 | 0.24 | 2.01 | | | | | | | | | | |
| Prostate | 8 | 13.95 | 0.57 | 0.24 | 1.04 | 3 | 4.4 | 0.68 | 0.13 | 1.67 | 6 | 2.94 | 2.04 | 0.73 | 4.00 |
| Kidney | 3 | 4.90 | 0.61 | 0.12 | 1.50 | | | | | | 3 | 1.03 | 2.91 | 0.55 | 7.14 |
| Bladder | 4 | 6.37 | 0.63 | 0.16 | 1.39 | | | | | | | | | | |
| Melanoma | 8 | 7.10 | 1.13 | 0.48 | 2.04 | 5 | 2.24 | 2.23 | 0.70 | 4.62 | | | | | |
| Skin | 3 | 3.25 | 0.92 | 0.17 | 2.26 | 2 | 1.02 | 1.96 | 0.18 | 5.62 | 2 | 0.68 | 2.94 | 0.28 | 8.43 |
| Nervous system | 18[a] | 6.74 | **2.67** | **1.58** | **4.05** | | | | | | 3 | 1.42 | 2.11 | 0.40 | 5.18 |
| Thyroid glands | 3 | 2.11 | 1.42 | 0.27 | 3.49 | | | | | | | | | | |
| Connective tissue | 2 | 1.25 | 1.60 | 0.15 | 4.59 | | | | | | | | | | |
| Lymphoma | 2 | 6.04 | 0.33 | 0.03 | 0.95 | | | | | | | | | | |
| Leukaemia | 3 | 3.99 | 0.75 | 0.14 | 1.84 | | | | | | | | | | |
| Other sites | 7 | 10.86 | 0.64 | 0.26 | 1.21 | 11 | 14.34 | 0.77 | 0.38 | 1.29 | 12 | 7.45 | 1.61 | 0.83 | 2.65 |

Bold type: 95% CI does not include 1.00. [a]There were 9 cases with age at diagnosis < 5, SIR 5.14, 95% CI 2.33–9.05.

**Table 4** SIR for childhood brain tumour by parental brain cancer

| Parental cancer | Age at diagnosis of astrocytoma | | | | | | | | | | Age at diagnosis of all brain cancer | | | | | | | | | |
|---|---|---|---|---|---|---|---|---|---|---|---|---|---|---|---|---|---|---|---|---|
| | < 5 | | | | | < 15 | | | | | < 5 | | | | | < 15 | | | | |
| | O | E | SIR | 95% CI | | O | E | SIR | 95% CI | | O | E | SIR | 95% CI | | O | E | SIR | 95% CI | |
| Astrocytoma | 3 | 0.83 | 3.61 | 0.68 | 8.86 | 7 | 3.23 | 2.17 | 0.86 | 4.07 | 4 | 1.88 | 2.13 | 0.55 | 4.72 | 10 | 6.55 | 1.53 | 0.73 | 2.62 |
| Meningioma | 4 | 0.39 | **10.26** | **2.67** | **22.77** | 7 | 1.63 | **4.29** | **1.70** | **8.07** | 4 | 0.89 | **4.49** | **1.17** | **9.98** | 7 | 3.30 | 2.12 | 0.84 | 3.98 |
| All brain tumours | 8 | 1.56 | **5.13** | **2.19** | **9.30** | 14 | 6.11 | **2.29** | **1.25** | **3.65** | 9 | 3.53 | **2.55** | **1.16** | **4.49** | 20 | 12.38 | 1.62 | 0.99 | 2.40 |

Bold type: 95% CI does not include 1.00.

**Table 5** SIR for cancer in siblings of a childhood brain tumour patient

| Cancer sites | Sib's age at diagnosis | | | | | | | | | |
|---|---|---|---|---|---|---|---|---|---|---|
| | < 15 | | | | | ⩾ 15 | | | | |
| | O | E | SIR | 95% CI | | O | E | SIR | 95% CI | |
| Colon | | | | | | 2 | 0.80 | 2.50 | 0.24 | 7.17 |
| Breast | | | | | | 4 | 3.39 | 1.18 | 0.31 | 2.62 |
| Testis | | | | | | 4 | 1.25 | 3.21 | 0.83 | 7.13 |
| Melanoma | | | | | | 2 | 2.25 | 0.89 | 0.08 | 2.54 |
| Nervous system | 3[a] | 1.85 | 1.62 | 0.31 | 3.97 | 7[b] | 1.97 | **3.55** | **1.41** | **6.66** |
| Endocrine glands | | | | | | 3 | 0.81 | 3.69 | 0.70 | 9.06 |
| Leukaemia | 2 | 1.81 | 1.11 | 0.10 | 3.18 | 2 | 0.88 | 2.27 | 0.21 | 6.49 |
| Other sites | 3 | 0.70 | 4.29 | 0.81 | 10.51 | 3 | 0.54 | **5.60** | **1.06** | **13.72** |

Bold type: 95% CI does not include 1.00. [a]2 sib-pairs had astrocytoma, 1 astrocytoma-ganglioneuroma. [b]1 sib-pair had astrocytoma, 1 astrocytoma-medulloblastoma, 1 medulloblastoma, 1 medulloblastoma- ependymoma, 1 neurinoma, 1 ependymoma-pinealoma and 1 astrocytoma-benign tumour (PAD missing).

*© 2000 Cancer Research Campaign* 

tumour was 5.13 (< 5 years) and 2.29 (Table 4). When both parents and offspring had astrocytoma the SIR was 3.61 (< 5 years) and 3.23 (< 15 years). There was a significant association of offspring astrocytoma with parental meningioma, SIRs being 10.26 and 4.29 when diagnosed before age 5 and 15, respectively. Only one parent of a brain tumour proband had a brain tumour in childhood. There were 29 cases of any second cancer in childhood brain tumour probands, of these seven had a parent with any cancer and two of these were brain tumour.

Sibling risks were calculated separately for siblings of childhood brain tumour probands when the sib was diagnosed before age 15 or in adult age (15–61 years, Table 5). SIR for brain tumour in the 3 siblings diagnosed before age 15 was 1.62 but it was 3.55 (95% CI, 1.41–6.66) for brain tumours presenting later. The types of nervous system cancers in the sib-pairs were heterogeneous as shown in the footnote; 3/10 pairs were astrocytomas.

### DISCUSSION

Childhood brain tumour is represented in many rare cancer syndromes of high risk (Draper et al, 1996), but hereditary effects have been ascribed only to some 4% of brain tumours (Bondy et al, 1991). A study on cancer in parents of childhood cancer probands found no increase in the risk of nervous system cancer between the two generations (Olsen et al, 1995); the only parental site that associated with nervous system cancer in offspring was rectal tumour in fathers but not in mothers. In the present material only 1.3% of childhood tumour cases had a parent with nervous system cancer. SIR for brain tumours in offspring of affected parents was 2.54 and 1.88, for diagnoses before age 5 or 15, respectively. We may compare this with the familial risk for nervous system cancer diagnosed in adult age in the Family-Cancer Database of 1.8 to 1.9 (Hemminki et al, 1998; Hemminki and Kyyronen, 1999). The sibling risk for nervous system cancer of 3.55 included siblings who were diagnosed for nervous system cancer in ages 15–61 years. Previous studies on siblings and twins also found moderate risks (Draper et al, 1977; Buckley et al, 1996).

There was an interesting difference in the familial risks by brain tumour subtypes. Parental nervous system cancers associated only with offspring astrocytomas, salivary gland cancers associated with medulloblastomas and colon cancers associated with ependymomas. All these findings are novel for population-based studies on childhood brain tumours. An aggregation of adult astrocytomas has recently been described from Sweden (Malmer et al, 1999). Li-Fraumeni syndrome features the occurrence of diverse gliomas (Sedlacek et al, 1998), and it is possible that some of the astrocytoma aggregates were due to this syndrome. The highest risk of offspring astrocytomas was found in combination with parental meningioma. This combination could be found in neurofibromatosis, particularly of type 2 (Huson, 1998). The association of brain and colon cancers is known in hereditary non-polyposis colorectal cancer (HNPCC) and in Turcot's syndrome but in both only individual cases of adult ependymoma have been described in the literature (Vasen et al, 1996; Paraf et al, 1997; Mullins et al, 1998). HNPCC is also associated with a high risk of endometrial cancer (Aarnio et al, 1999) and in our material there were two ependymoma patients whose mothers had endometrial cancer (SIR 2.17, 95% CI, 0.20–6.23).

The present data showed distinct patterns of familial risks for childhood brain tumour including brain astrocytoma-meningioma between the two generations. Parental colon cancer was associated with offspring ependymoma, and parental salivary gland cancers with offspring medulloblastoma. Some of these aggregations may belong to recognized cancer syndromes but genotyping would be required for confirmation. Risk of nervous system cancer to siblings from brain tumour probands was noted when siblings were followed to adult age.

### ACKNOWLEDGEMENTS

Supported by the King Gustaf V Jubilee Fund, The Swedish Council for Planning and Coordination of Research and The Cancer Fund.

### REFERENCES

Aarnio M, Sankila R, Pukkala E, Salovaara R, Aaltonen LA, de la Chapelle A, Peltomaki P, Mecklin JP and Jarvinen HJ (1999) Cancer risk in mutation carriers of DNA-mismatch-repair genes. *Int J Cancer* **81**: 214–218.

Bondy ML, Lustbader ED, Buffler PA, Schull WJ, Hardy RJ and Strong LC (1991) Genetic epidemiology of childhood brain tumors. *Genet Epidemiol*, **8**: 253–267.

Buckley JD, Buckley CM, Breslow NE, Draper GJ, Roberson PK and Mack TM (1996) Concordance for childhood cancer in twins. *Med Pediatr Oncol* **26**: 223–229.

Draper GJ, Heaf MM and Kinnier Wilson LM (1977) Occurrence of childhood cancers among sibs and estimation of familial risks. *J Med Genet* **14**: 81–90.

Draper GJ, Kroll ME and Stiller CA (1994) Childhood cancer. *Cancer Surv* **20**: 493–517.

Draper GJ, Sanders BM, Lennox EL and Brownbill PA (1996) Patterns of childhood cancer among siblings. *Br J Cancer* **74**: 152–158.

Esteve J, Benhamou E and Raymond L (1994) Statistical methods in cancer research. Volume IV. Descriptive epidemiology. *IARC Sci Publ* **128**: 1–302.

Fearon ER (1997) Human cancer syndromes: clues to the origin and nature of cancer. *Science* **278**: 1043–1050.

Hemminki K and Dong C (2000) Familial relationships in thyroid cancer by histopathological type. *Int J Cancer* **85**: 201–205.

Hemminki K and Kyyronen P (1999) Parental age and risk of sporadic and familial cancer in offspring: implications for germ cell mutagenesis. *Epidemiology* **10**: 747–751.

Hemminki K and Vaittinen P (1998a) Familial breast cancer in the family-cancer database. *Int J Cancer* **77**: 386–391.

Hemminki K and Vaittinen P (1998b) National database of familial cancer in Sweden. *Genet Epidemiol* **15**: 225–236.

Hemminki K and Vaittinen P (1999) Familial cancers in a nationwide family cancer database: age distribution and prevalence. *Eur J Cancer* **35**: 1109–1117.

Hemminki K, Vaittinen P and Kyyronen P (1998) Age-specific familial risks in common cancers of the offspring. *Int J Cancer* **78**: 172–175.

Hemminki K, Kyyronen P and Vaittinen P (1999) Parental age as a risk factor of childhood leukemia and brain cancer in offspring. *Epidemiology* **10**: 271–275.

Hjalmars U, Kulldorff M, Wahlqvist Y and Lannering B (1999) Increased incidence rates but no space-time clustering of childhood astrocytoma in Sweden, 1973–1992: a population-based study of pediatric brain tumors. *Cancer* **85**: 2077–2090.

Huson S (1998) Neurofibromatosis type 1: historical perspective and introductory overview. In: *Neurofibromatosis Type 1*, Upadhyaya M and Cooper D (eds) pp. 1–20. BIOS: Oxford.

IARC (1998) *International Incidence of Childhood Cancer*. Vol II. IARC Sci Publ No. 144. IARC: Lyon.

Kleihues P and Cavenee W (2000) *Tumors of the nervous system*. IARC: Lyon.

Li F (1995) Phenotypes, genotypes, and interventions for hereditary cancers. *Cancer Epidemiology Biomarkers Prev* **4**: 579–582.

Linet MS, Ries LA, Smith MA, Tarone RE and Devesa SS (1999) Cancer surveillance series: recent trends in childhood cancer incidence and mortality in the United States. *J Natl Cancer Inst* **91**: 1051–1058.

Little J (1999) *Epidemiology of Childhood Cancer*. Vol 149. IARC Sci Publ. IARC: Lyon.

Little MP, de Vathaire F, Shamsaldin A, Oberlin O, Campbell S, Grimaud E, Chavaudra J, Haylock RG and Muirhead CR (1998) Risks of brain tumour

 © 2000 Cancer Research Campaign following treatment for cancer in childhood: modification by genetic factors, radiotherapy and chemotherapy. *Int J Cancer* **78**: 269–275.

Malmer B, Gronberg H, Bergenheim AT, Lenner P and Henriksson R (1999) Familial aggregation of astrocytoma in northern Sweden: an epidemiological cohort study. *Int J Cancer* **81**: 366–370.

Mullins KJ, Rubio A, Myers SP, Korones DN and Pilcher WH (1998) Malignant ependymomas in a patient with Turcot's syndrome: case report and management guidelines. *Surg Neurol* **49**: 290–294.

Narod SA, Stiller C and Lenoir GM (1991) An estimate of the heritable fraction of childhood cancer. *Br J Cancer* **63**: 993–999.

Olsen JH, Boice JD, Jr, Seersholm N, Bautz A and Fraumeni JF, Jr (1995) Cancer in the parents of children with cancer [see comments]. *N Engl J Med* **333**: 1594–1599.

Paraf F, Jothy S and Van Meir EG (1997) Brain tumor-polyposis syndrome: two genetic diseases? *J Clin Oncol* **15**: 2744–2758.

Salminen E, Pukkala E and Teppo L (1999) Second cancers in patients with brain tumours – impact of treatment. *Eur J Cancer* **35**: 102–105.

Sankila R, Olsen JH, Anderson H, Garwicz S, Glattre E, Hertz H, Langmark F, Lanning M, Moller T and Tulinius H (1998) Risk of cancer among offspring of childhood-cancer survivors. Association of the Nordic Cancer Registries and the Nordic Society of Paediatric Haematology and Oncology [see comments]. *N Engl J Med* **338**: 1339–1344.

Sedlacek Z, Kodet R, Poustka A and Goetz P (1998) A database of germline p53 mutations in cancer-prone families. *Nucleic Acids Res* **26**: 214–215.

Vasen HF, Sanders EA, Taal BG, Nagengast FM, Griffioen G, Menko FH, Kleibeuker JH, Houwing-Duistermaat JJ and Meera Khan P (1996) The risk of brain tumours in hereditary non-polyposis colorectal cancer (HNPCC). *Int J Cancer* **65**: 422–425.

Zahm SH and Devesa SS (1995) Childhood cancer: overview of incidence trends and environmental carcinogens. *Environ Health Perspect* **103 Suppl 6**: 177–184.

*© 2000 Cancer Research Campaign*
